# Novel Gait Training with a Hybrid Assistive Limb Improved Delayed Progressive Spastic Paraplegia After a Lightning Strike

**DOI:** 10.3390/jcm14030967

**Published:** 2025-02-03

**Authors:** Yuichiro Soma, Shigeki Kubota, Hideki Kadone, Yukiyo Shimizu, Kousei Miura, Yasushi Hada, Yoshiyuki Sankai, Masashi Yamazaki

**Affiliations:** 1 Division of Regenerative Medicine for Musculoskeletal System, Institute of Medicine, University of Tsukuba, Tsukuba 305-8577, Japan; skubota@tsukuba-seikei.jp; 2 Department of Orthopaedic Surgery, Institute of Medicine, University of Tsukuba, Tsukuba 305-8577, Japan; kmiura@tsukuba-seikei.jp (K.M.); masashiy@md.tsukuba.ac.jp (M.Y.); 3 Center for Innovative Medicine and Engineering (CIME), University of Tsukuba, Tsukuba 305-0821, Japan; kadone@ccr.tsukuba.ac.jp (H.K.); sankai@golem.iit.tsukuba.ac.jp (Y.S.); 4 Department of Rehabilitation Medicine, Institute of Medicine, University of Tsukuba, Tsukuba 305-8577, Japan; shimiyukig@md.tsukuba.ac.jp (Y.S.); y-hada@md.tsukuba.ac.jp (Y.H.)

**Keywords:** hybrid assistive limb, lightning strike, delayed progressive paraplegia, neurorehabilitation, spasticity

## Abstract

**Background/Objectives**: A 68-year-old man presented with progressive walking difficulty that developed into spastic paraplegia. This condition was a long-term consequence of a lightning strike injury sustained at the age of 22 years. His symptoms progressively deteriorated, eventually requiring double crutches for ambulation at approximately 40 years of age. A physical evaluation prior to hybrid assistive limb (HAL) training revealed a T10 level neurological injury and an American Spinal Cord Injury Association impairment scale grade D. Here, we aimed to evaluate the therapeutic effects of novel gait training with an HAL in this patient with chronic and progressive neural damage caused by a lightning strike. **Methods**: The HAL training program is composed of two sections. In the first section, one month of gait training with HAL was conducted across 10 sessions, with 2–3 sessions weekly. The second section followed 6 months later. A final evaluation was performed three months after the second section. **Results**: Electromyographic and kinematic evaluation showed that the HAL gait training inhibited abnormal antagonistic muscle activation in his lower extremities, especially after the first section. Our results collectively indicate that the repeated HAL gait training improved the gait pattern of this patient. **Conclusions**: Our results suggest that HAL gait training may improve the gait pattern in patients with delayed progressive spastic paraplegia, as observed in this case. In addition, a longer intervention period is recommended to facilitate better adaptation to HAL training. Hence, neurorehabilitation with an HAL could be an innovative treatment approach for delayed progressive spastic paraplegia.

## 1. Introduction

Many patients affected by lightning strikes are healthy young individuals participating in outdoor recreational activities [1,2]. Lightning-related neurological damage typically involves the central nervous system rather than the peripheral nervous system [3]. A previous study suggested that neurological syndromes resulting from lightning-related damage could be categorized into four groups: immediate and transient; immediate and prolonged or permanent; delayed, traumatic lesions due to falls; and blast effects [3]. Although delayed spinal cord injuries following high-voltage electrical burns are not typically life-threatening, complete recovery is rare and leads to significant morbidity [4]. Another study comprehensively analyzed the clinical characteristics, radiographic findings, and clinical outcomes of patients who experienced delayed spinal cord injuries after electrical burns. In cases where the entry sites were located in the head and neck area, paraplegia was observed when the exit sites were confined to the lower extremities [5].

Delayed spinal cord injury (SCI) may manifest a few days to several months following electrical trauma, often resulting in incomplete and permanent damage [6]. The primary mechanism involves the heating effect, which induces cell membrane rupture through electroporation, leading to delayed SCI. Additionally, ischemic injury, stemming from endothelial damage or thrombosis, is a major contributor to this condition. The anterior spinal cord, characterized by its rich vascular distribution, is particularly vulnerable. Damage to this region frequently disrupts motor tracts, resulting in the deterioration of motor function [7]. From a neurorehabilitation perspective, therapeutic principles for addressing lightning- or electrical trauma-induced motor dysfunction align closely with those employed for more common injury types. The primary goal is to optimize functional recovery by targeting specific impairments unique to an individual [8]. Recently, we encountered a patient with an SCI caused by a lightning strike who experienced progressive worsening of spastic paraplegia more than 30 years after the initial injury. This case represents an extremely rare pathological condition. Previous studies have demonstrated the efficacy of neurorehabilitation for neural damage caused by lightning strikes; however, they focused on patients within a maximum of 1 year post-injury [3,4,5]. To the best of our knowledge, there are no reports on neurorehabilitation for chronic-stage lightning strike-induced spinal cord damage presenting severe spastic paraplegia. There is a need for the development of novel neurorehabilitation approaches for patients with severe spastic palsy [9]. Therefore, we implemented a novel neurorehabilitation procedure for this patient.

The hybrid assistive limb (HAL) robot is a wearable exoskeleton cyborg. It provides real-time assistance for walking and limb movement using actuators mounted on the bilateral hip and knee joints [10,11]. Studies have been conducted on HAL training in patients with spinal and other related disorders. Motor function restoration is believed to induce the structural reorganization of neuronal circuits in the injured spinal cord [12,13]. However, the pathological conditions following lightning and electrical trauma differ from those following spinal cord injuries or disorders. For instance, spinal cord stroke arises from the abrupt disruption of blood supply to the spinal cord, resulting in ischemia, infarction, acute spinal cord dysfunction, and related clinical neurological deficits. These deficits are associated with the affected territories of the anterior spinal artery and two posterior spinal arteries [14]. A previous study reported the effectiveness of HAL in patients with spinal cord infarction, a condition characterized by spinal cord ischemia [15]. The study demonstrated the utility of HAL in gait rehabilitation without exacerbating spasticity. Here, we applied HAL gait training for a patient who sustained neural damage due to a lightning strike, marking the first instance of such an application in this specific context. Our objectives were three-fold: to ascertain the suitability of HAL training for a patient with neural damage resulting from a lightning strike, review the clinical presentation of a patient in terms of gait kinematics and kinetics, and evaluate the repeated intervention effects of HAL. We hypothesize that HAL training can enhance ambulatory capabilities by employing a mechanism that combines voluntary driving and normalized motion assistance facilitated by an external device (HAL). This integrated approach can potentially establish a proprioceptive feedback loop, which is especially beneficial for patients with lesions affecting the sensory pathways. As a result, this feedback loop may reduce spastic co-contraction within the lower limbs, ultimately optimizing walking ability. Accordingly, we applied HAL gait training to our patient who sustained neural damage from a lightning strike and exhibited delayed, progressive severe spastic paraplegia. As the condition is exceptionally rare, detailing the application of HAL training for managing it holds high value.

## 2. Materials and Methods

### 2.1. Patient Information

**Figure 1:**
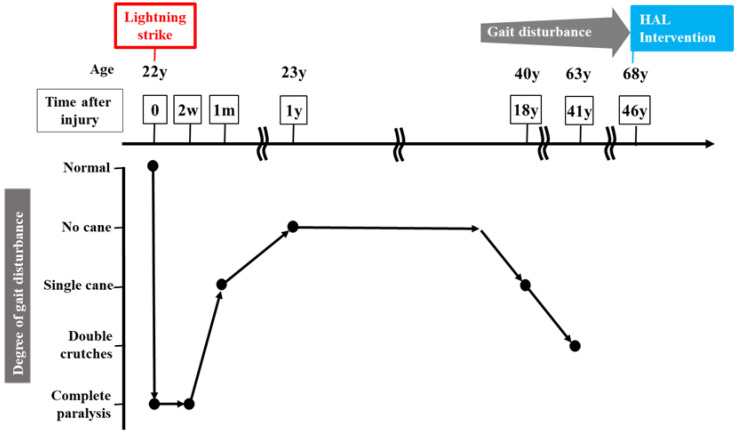
Progression of the patient’s symptoms following the lightning strike injury and the timing of the initiation of hybrid assistive limb gait training.

**Figure 2:**
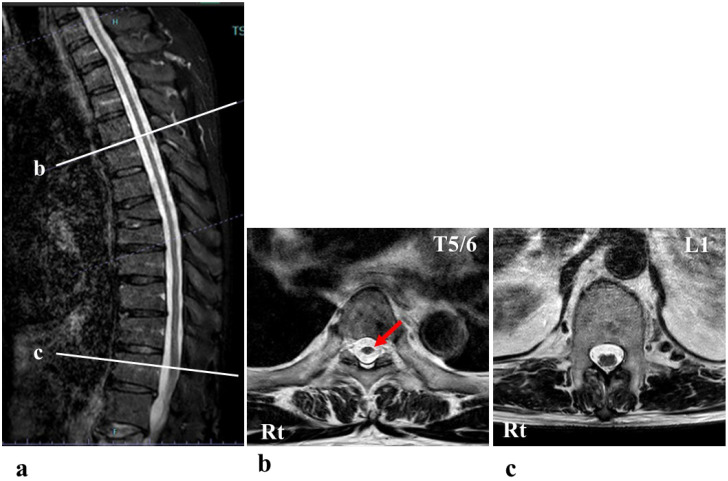
Mid-sagittal section (**a**) and axial sections at the T5/6 level (**b**) and L1 level (**c**) of the T2-weighted magnetic resonance image showed that his thoracic spinal cord was slightly atrophic (arrow in (**b**)).

A 68-year-old man presented with progressive walking difficulties, which progressed to spastic paraplegia that began to worsen around the age of 40 years (Figure 1). This condition resulted from a high-tension electrical injury sustained at 22 years of age. He was struck by lightning while trekking in the Northern Japanese Alps. The lightning entered his body through the head and neck areas and exited through the lower extremities. Immediately after the injury, the patient became completely paralyzed. Two weeks post-injury, the patient began to recover gradually, and by 1 month post-injury, he was able to walk with a single T-cane. At 1 year post-injury, the patient regained the ability to walk without a cane, and this relatively stable condition persisted for more than 30 years. However, around the age of 40 years, his gait disturbance progressively worsened, requiring the use of a T-cane for mobility. The gait disturbance continued to deteriorate, and around the age of 63 years, the patient required double crutches for walking. At the age of 68 years, 46 years after the initial injury, the patient was admitted to our institution with a documented history of severe and worsening spastic paraplegia, prompting the initiation of HAL gait training. At the time of admission, his physical examination revealed that activities of daily living were substantially impaired because of severe spasticity in both lower limbs. He could only manage to stand up from his wheelchair with considerable support. While the patient was able to walk short distances using two crutches, his gait was unsteady and awkward. Prior to HAL training, the patient’s physical evaluation revealed a neurological level of injury of T10. According to the American Spinal Cord Injury Association impairment scale (AIS), the patient was classified as grade D [16]. The motor score, as per the International Standards for Neurological and Functional Classification of Spinal Cord Injury (ISNCSCI), was 76 points (right: 37 points; left: 39 points). The sensory score was 108 points, derived from both light touch and pinprick tests (right: 52 points; left: 56 points) [16,17]. The patient’s deep tendon reflexes were hyperactive in the lower extremities. Furthermore, his Walking Index for Spinal Cord Injury (WISCI) II score was 11 points [18,19]. His functional independence measure (FIM) motor score was 78 points [20]. Magnetic resonance imaging (MRI) showed that his thoracic spinal cord was slightly atrophic (Figure 2). Notably, there was no evidence of spinal cord compression or abnormal signal intensity within the spinal cord. Despite these findings, the patient reported a substantial progression of walking difficulties and challenges with daily activities over the past 20 years, and this was attributed to worsening spastic paralysis in the lower limbs. Previous reports suggest that there is no specific imaging modality to reliably diagnose SCI caused by electrical trauma as imaging findings are often unremarkable [21]. In cases where SCI symptoms are present, MRI and other imaging test results typically appear normal. Consequently, the diagnosis of neurological injury in such cases relies heavily on clinical findings and the progression of symptoms rather than imaging results [21]. Moreover, regarding the differential diagnosis in this case, the possibility of disuse atrophy was ruled out owing to the lack of clear muscle atrophy and the fact that spasticity in the lower limbs had intensified [22].

### 2.2. HAL Gait Training

The patient experienced substantial challenges with gait performance during daily activities. Progressive spasticity notably affected his lower limb kinematics, leading to abnormalities such as knee hyperextension and compensatory toe clearance during walking. Considering these issues, addressing the spastic gait pattern and preventing further deterioration in daily functional capabilities, including standing and ambulation, became a priority. The patient felt discomfort and difficulty in response to the worsening challenges in daily activities. Therefore, a combined approach involving conventional rehabilitation and HAL gait training was considered the most effective strategy to efficiently tackle these issues [12].

**Figure 3:**
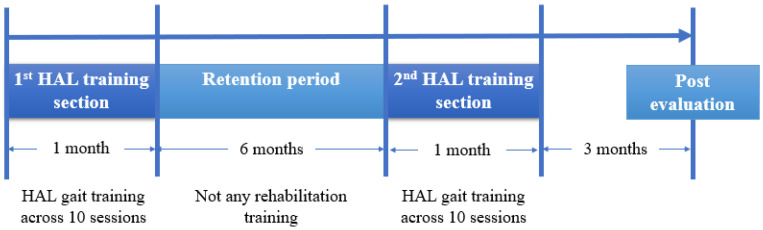
Schematic representation of the hybrid assistive limb (HAL) gait training program used in this study. The program comprised 1 month of gait training using HAL conducted across 10 sessions, ranging between 2 and 3 sessions weekly, a 6-month retention period during which the patient did not undergo any rehabilitation training, another month of HAL training, and an evaluation 3 months after the 2nd HAL training section.

**Figure 4:**
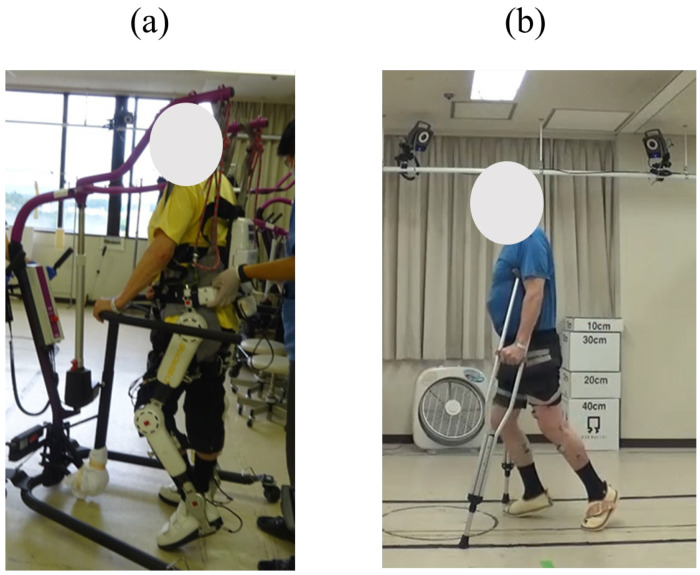
(**a**) Photograph of the hybrid assistive limb exoskeleton featuring the All-in-One Walking Trainer with its harness system. This setup was used during gait training sessions to prevent falls and provide support for the safe movement of the patient. (**b**) For the 10 m walking test, the patient was instructed to walk on a flat surface at a comfortable pace while using double crutches.

The HAL gait training program consisted of one month of alternating phases of HAL-assisted robotic gait training and retention periods. The 2nd HAL training section commenced 6 months after the 1st HAL training section, with an evaluation conducted three months after the 2nd HAL training section (Figure 3). One of the primary objectives of this study was to evaluate the effects of repeated HAL training for gait performance, especially spastic gait pattern. To ensure effective training, a team consisting of two therapists and an assistant managed the attachment and detachment of the HAL exoskeleton suit. Additionally, an engineer was responsible for conducting gait movement analysis. All HAL training sessions, which included activities such as standing, sitting, and walking, were supplemented with an All-in-One Walking Trainer (Ropox A/S; Naestved, Denmark) to prevent potential falls. For added safety and support, a harness was used to deliver body weight support (Figure 4a). Under the guidance of a physical therapist, HAL training sessions were tailored with adjustable body weight support. This adjustment aimed to provide a comfortable gait movement for the patient, considering the patient’s posture, ensuring sufficient knee extension during the stance phase, and maintaining foot clearance throughout the swing phase. Typically, a session lasted 60 min, which covered both the fitting of the exoskeleton and the subsequent evaluation. Core gait training time within each session was 20 min. In this case, we primarily used the Cybernic Voluntary Control (CVC) mode of the HAL exoskeleton. This mode enables the operator to adjust the level of physical support to optimize patient comfort. Decisions regarding walking distance, walking speed, and the amount of body weight support during each HAL training session were informed by factors such as the patient’s vital signs, fatigue level, and motivation. Additionally, conventional rehabilitation was carried out by a physiotherapist four times a week, with each session lasting 40 min.

### 2.3. Measurements

HAL gait training was divided into two sections, with the 2nd section commencing 6 months after the first. Throughout the study period, we conducted assessments to identify any adverse events that could be associated with HAL training. Adverse events were defined as any unexpected or unfavorable symptoms, diseases, or signs of such conditions, including clinical laboratory data abnormalities. The monitored adverse events included death, life-threatening conditions, disability or incapacity, potential disability, and hospitalization.

Specific metrics were evaluated before the start of the first and fifth HAL sessions (pre-1 session and pre-5 session) and after the conclusion of all HAL sessions (post-10 session). The walking metrics, including walking speed, step length, and cadence, were assessed using a 10 m walking test. These parameters were evaluated during the fifth session of HAL training. Additionally, the ISNCSCI motor scores, WISCI-II, FIM motor score, and walking parameters were reassessed 3 months after the second HAL training session. During the 10 m walking test, the patient was instructed to walk at a comfortable pace on a flat surface while using double crutches (Figure 4b). Walking time was measured using a handheld stopwatch, and these data were used to calculate walking speed. Step length was derived by counting the number of steps taken between the start and finish lines. Cadence was determined by dividing the total number of steps by the walking time, yielding steps per minute. The total HAL gait training distance during the 20 min core gait training time was recorded in all sessions of the 1st and 2nd sections. In addition, the walking speed, step length, and cadence were measured during HAL training at the pre-1, pre-5, and post-10 sessions.

### 2.4. Joint Angle Kinematics Data Analysis

To assess whether the HAL intervention affected lower limb movement and gait kinematics, a 10 m walking test was conducted, and the results were compared using a motion capture system (Vicon MX with 16 T20S cameras, 100 Hz, plug-in gait marker set, Oxford, UK). The test was performed at the pre-1 and post-10 sessions in the 1st and 2nd HAL sections, respectively, without the participant wearing the HAL suit [23,24]. Auto-reflective markers were placed on the following anatomical landmarks according to the plug-in gait marker set: the anterior superior iliac spine, posterior superior iliac spine, lower lateral 1/3rd surface of the thigh, flexion-extension axis of the knee, lower lateral 1/3 surface of the shank, head of the second metatarsal bone of the toe, lateral malleolus of the ankle, and posterior peak of the heel calcaneus. These markers enabled detailed analysis of the gait parameters to evaluate the effects of HAL training on walking biomechanics.

### 2.5. Electromyography Evaluation

Electromyography (EMG) was conducted during the 10 m walking test without the participant wearing the HAL suit at the pre-1 and post-10 sessions in the 1st and 2nd HAL sections, respectively. EMG sensors were placed bilaterally on the muscles around the hip, knee, and ankle joints, specifically targeting the rectus femoris, gluteus maximus, hamstrings, tibialis anterior, and gastrocnemius muscles. The activity of each muscle was recorded using a TrignoTM Lab Wireless EMG System (Delsys, Inc., Boston, MA, USA). Data were sampled at a frequency of 2 kHz and synchronized with the motion capture system to analyze muscle activation patterns and assess changes in neuromuscular function throughout the training sessions.

### 2.6. Data Analysis

For each extracted step cycle, the maximum and minimum angles at the hip, knee, and ankle joints, as well as the maximum anterior and posterior angles of the pelvis, were calculated to assess the joint range of motion. The kinematic profile of each lower extremity for each step cycle was normalized to the total duration of the cycle and averaged across multiple cycles for a representative analysis. The EMG data were filtered using a 30–400 Hz bandpass filter with scripts in MATLAB 8.2 (MathWorks, Natick, MA, USA) then rectified and locally integrated using a 200 ms moving window to create an integrated EMG profile. The joint angle and integrated EMG profiles for each step were time-normalized to 101 points, with 0% corresponding to the heel strike that initiated the cycle and 100% representing the subsequent heel strike that terminated the cycle. This approach provided a time-aligned, detailed view of joint kinematics and muscle activation patterns throughout the gait cycle, allowing for comparison between pre- and post-HAL intervention conditions.

The walking speed, stride length, cadence, and toe lift height were compared between conditions with and without HAL during the fifth session in both the 1st and 2nd sections. Additionally, the variability in stride length, cadence, and toe lift height between the conditions with HAL and without HAL was assessed using the coefficient of variation (CV). The CV was calculated as the standard deviation divided by the mean and multiplied by 100 for each measurement [25].

### 2.7. Informed Consent Statement

The patient received a personalized explanation of the study, participation, and data usage before signing an informed consent form. The patient agreed to participate in this study and provided informed consent in both oral and written form.

## 3. Results

### 3.1. Gait Training with the Application of HAL

Careful monitoring was performed to evaluate any severe fatigue or pain associated with HAL training both immediately after each session and the following day. Except for mild fatigue reported the following day, no serious adverse events or instances of resistance to the HAL training were observed during any session.

**Figure 5:**
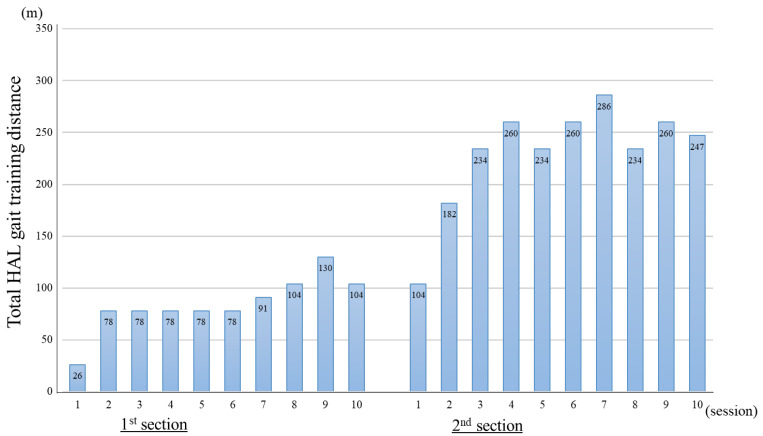
Total hybrid assistive limb gait training distance during the 20 min core gait training time was recorded in all sessions of the first and second sections.

Figure 5 shows the total gait training distance for each session during HAL training time. In the first section, the distance for session 1 was 24 m. In the second section, the distance increased to 247 m in the 10th session (Video S1).

### 3.2. Pre-Post-Comparison: 10 m Walking Test

Specific metrics, including the ISNCSCI motor score, WISCI-II score, FIM motor score, and walking parameters, are presented in Table 1. Slight changes were observed regarding the other outcomes, such as the ISNCSCI motor score (first HAL training, 76–79 points; second HAL training, 73–77 points; 3 months after second HAL training, 77 points), FIM motor score (first HAL training, 78–81 points; second HAL training, 82–83 points; 3 months after second HAL training, 83 points), and WISCI-II score (first HAL training, 11 points; second HAL training, 11–12 points; 3 months after second HAL training, 11 points).

In the 10th session of the first HAL training section, the walking speed with HAL was higher than that without HAL (non-HAL: 7.8 m/min, HAL: 10.7 m/min). In the second HAL training section, the walking speed and stride length with HAL were higher across all sessions than those without HAL. For walking speed: at session 1, non-HAL: 10.5 m/min, HAL: 11.7 m/min; at session 5, non-HAL: 10.2 m/min, HAL: 15.3 m/min; and at session 10, non-HAL: 10.6 m/min, HAL: 19.0 m/min. For stride length: at session 1, non-HAL: 0.35 m, HAL: 0.46 m; at session 5, non-HAL: 0.37 m, HAL: 0.5 m; and at session 10, non-HAL: 0.34 m, HAL: 0.55 m.

In the second HAL training section, all walking parameters improved compared to those in the first section. For instance, changes in walking speed were observed. In the first HAL section, walking speed decreased from 8.7 m/s at pre-session 1 to 7.8 m/s after 10 sessions. In the second HAL, it increased from 10.5 m/s at pre-session 1 to 10.6 m/s after 10 sessions. Stride length also changed, with the first HAL training decreasing from 0.34 m at pre-session 1 to 0.31 m after 10 sessions, while that in the second HAL training decreased from 0.35 m at pre-session 1 to 0.34 m after 10 sessions. Cadence showed similar patterns, decreasing from 25.7 cycles/min at pre-session 1 to 25.4 cycles/min after 10 sessions in the 1st HAL training and increasing from 30.1 cycles/min at pre-session 1 to 30.7 cycles/min after 10 sessions in the 2nd HAL training. Furthermore, these improvements in walking parameters were retained 3 months after the second HAL training session. The video shows the patient’s gait before and after the first and second HAL sections (Video S2).

**Figure 6:**
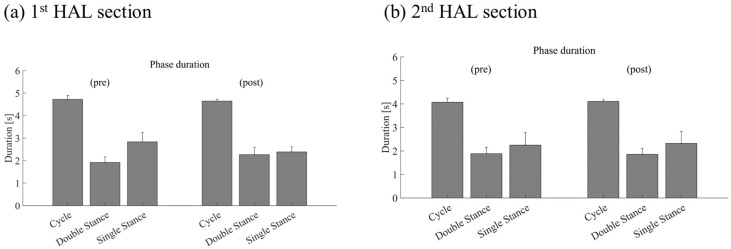
Profiles of the gait cycle time, both-leg support time, and one-leg support time pre- and post-assessment in the first (**a**) and second (**b**) hybrid assistive limb (HAL) sections.

In terms of the swing phase movement, we determined the cycle time, both-leg support time, and one-leg support time pre- and post-assessment in the first and second HAL sections. The cycle time was shortened, and the one-leg support time increased slightly in the second HAL section between the pre- and post-assessments (Figure 6a,b).

### 3.3. Pre-Post-Comparison: Joint Angle Kinematics

**Figure 7:**
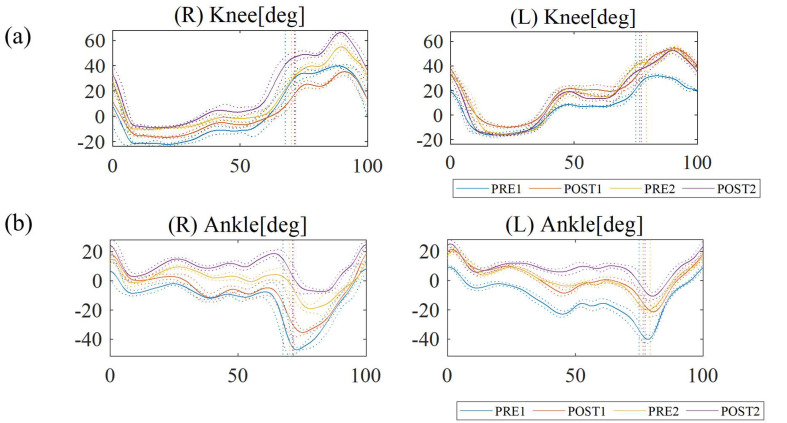
Temporal profile of the angular position of the right and left knee (**a**) and ankle joints (**b**) over the gait cycle, measured without the hybrid assistive limb (HAL) before session 1 (PRE) and after session 10 (POST) in the first (PRE1, POST1) and second HAL training sections (PRE2, POST2). Vertical lines indicate the moment of toe lift.

In the second HAL training section, the knee joint angles demonstrated greater flexion of the knee joint motion during the stance and swing phases than those in the first HAL training section (Figure 7a). The maximum dorsiflexion angles of the left and right ankle joint during the terminal swing phase to initial contact increased post-10 sessions (Figure 7b), demonstrating even greater angles in the second HAL training section than the first.

### 3.4. Muscular Activity Changes Observed After Pre-Post-Assessment Comparison

**Figure 8:**
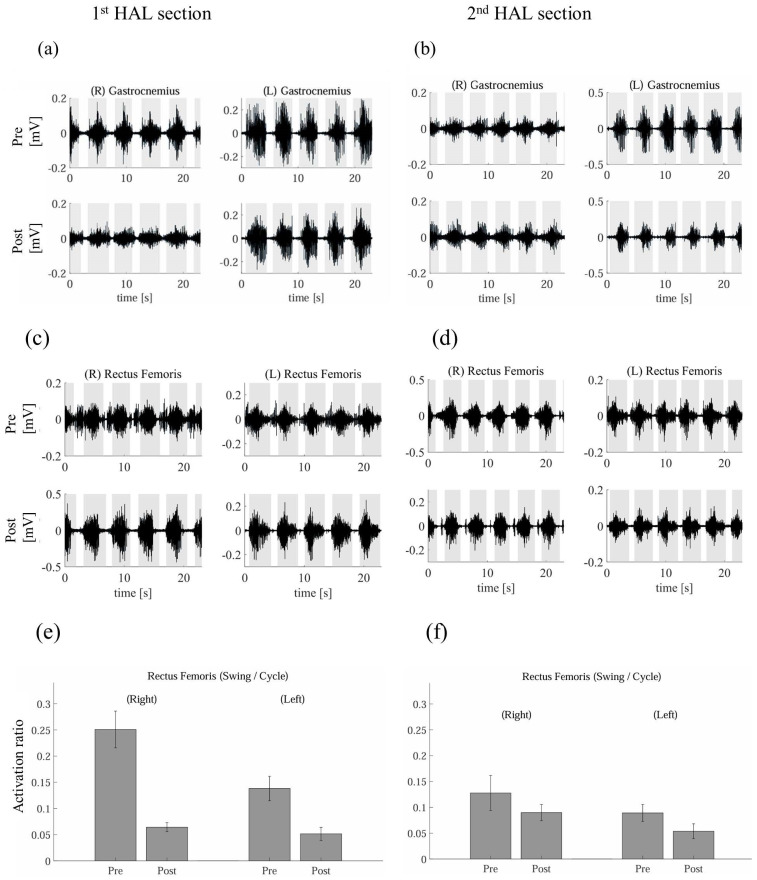
Surface electromyographs of the right and left gastrocnemius for the first (**a**) and second hybrid assistive limb (HAL) sections (**b**), pre and post-assessment and the right and left rectus femoris for the first (**c**) and second HAL sections (**d**). Gray bars indicate the stance phase. Also shown is the ratio of muscular activities during the swing phase pre- and post-assessment in the first (**e**) and second HAL sections (**f**).

Figure 8 shows the gastrocnemius EMG for the first and second HAL sessions both pre and post-assessment. The results indicated decreased activity in the second session compared to the activity in the first. In the first section, the activities of both limbs decreased post-assessment compared to those pre-assessment (Figure 8a). In the second section, left gastrocnemius activity decreased post-assessment compared to that pre-assessment (Figure 8b). Figure 8c,d show the rectus femoris EMG for the first and second HAL sections pre- and post-assessment. The results indicated decreased activity in the second section compared to the first. In addition, the activation ratio of the rectus femoris from the EMG data showed differences between pre- and post-assessment in the first and second HAL sections. Both sessions showed decreased activation during the swing phase (Figure 8e,f).

### 3.5. Gait Changes in HAL Sessions

**Figure 9:**
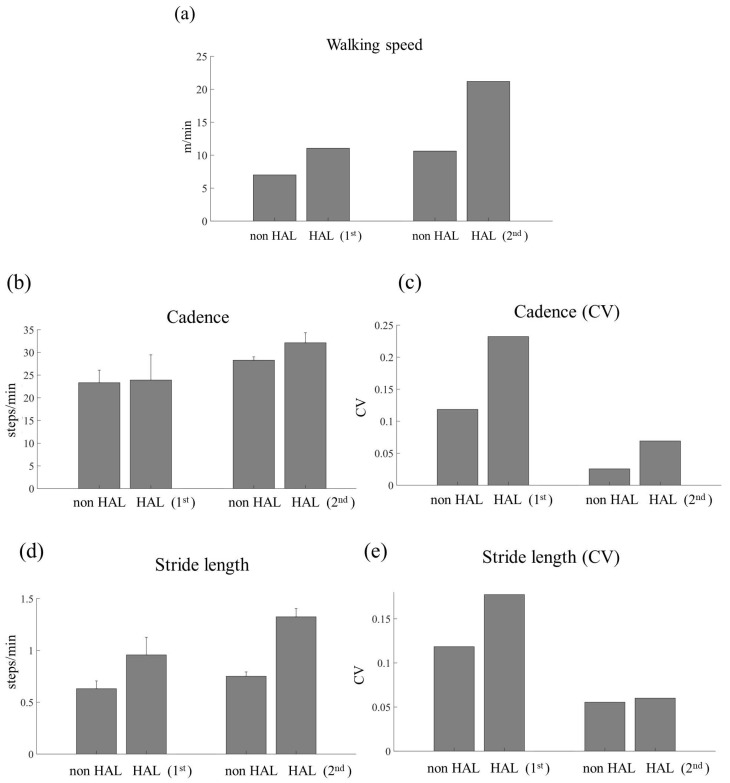

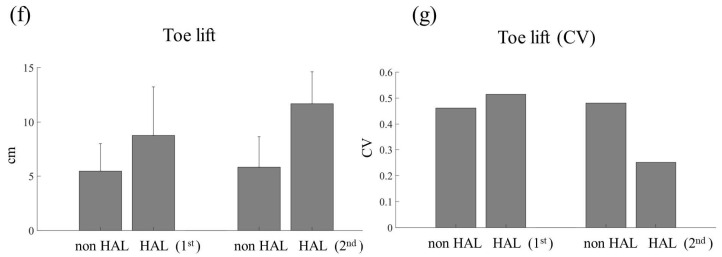
Walking speed with the hybrid assistive limb (HAL) and without HAL (non-HAL) during session 5 of the first section (first) and the second section (second) (**a**). The cadence with HAL and without HAL during session 5 of the first section and the second section (**b**) and the variability in the cadence between the conditions with HAL and without HAL were compared using the coefficient of variation (CV) (**c**). The stride length with HAL and without HAL during session 5 of the first section and the second section (**d**) and the variability in stride length between the conditions with HAL and without HAL were compared using the CV (**e**). The toe lift with HAL and without HAL during session 5 of the first section and the second section (**f**), the variability in the toe lift between the HAL and without HAL was compared using the CV (**g**).

The walking performance with and without HAL during the fifth session of the first and second sections and the walking speed are shown in Figure 9a. Additionally, variability in stride length, cadence, toe lift height, and their CV between walking with and without HAL are illustrated in Figure 9b–g. The results indicated that, in both the first and second sections, the walking speed with HAL was higher than that without HAL. Notably, the gait speed with HAL in the first section was comparable to that without HAL in the second section. Furthermore, in the second section, the difference in walking speed between conditions with HAL and without HAL was more pronounced.

The walking parameter results, including stride length, cadence, and toe lift height, were higher with HAL than those without HAL. Furthermore, in the second section, the differences in these parameters between the conditions with HAL and without HAL became more distinct. Regarding the CV, the variability in stride length and cadence decreased in the second section compared to that in the first section. However, in the second section, toe lift height with HAL decreased compared to that without HAL.

### 3.6. Patient-Reported Outcomes

In the first training section, the patient reported that he could stand more easily due to the decreased spasticity in the lower limbs compared with his condition before starting HAL training. Additionally, lifting objects while standing became easier, with the sensation of foot-lifting experienced during HAL training seemingly persisting. In the second section, the patient reported an increase in walking speed compared with that of before completing HAL training and the ability to maintain this improvement. Regarding HAL training, during the second section, the patient experienced reduced walking fatigue and demonstrated a more positive attitude toward the training compared with the first section. Based on this experience, the patient wishes to receive HAL training regularly as a form of ongoing treatment.

## 4. Discussion

To our knowledge, this study presents the first report on the feasibility and safety of HAL training in patients with spinal cord damage resulting from lightning and electrical trauma. Previous research has documented the feasibility and efficacy of HAL training in the rehabilitation of patients with impaired walking due to various conditions, such as musculoskeletal disorders, SCI, stroke, knee joint disease, and brachial plexus injury [26,27,28,29]. Throughout this year-long study, which included two rounds of HAL training, we meticulously evaluated the suitability of HAL training for this patient by monitoring potential adverse events and assessing its effects on clinical outcomes. Notably, no adverse events were observed throughout the study, confirming the feasibility and safety of HAL training for this patient.

The pathological condition involves the impairment of both upper and lower motor neurons. This finding is similar to what is observed in amyotrophic lateral sclerosis (ALS). ALS is a fatal motor neuron disease characterized by the degeneration of both upper and lower motor neurons, leading to muscle weakness, dysphagia, and respiratory disorders [30,31]. A previous study reported that gait function, including the 2 min and 10 m walk tests, improved after HAL training, suggesting that HAL may be effective in ameliorating and preserving gait ability in patients with ALS [32,33]. In contrast, the results of this study did not show improvement in the 10 m walking test using crutches after each HAL training section. Conducting the gait analysis using a walker (+harness) during HAL training may have been beneficial since it was used in a previous HAL study for ALS.

A previous study highlighted the potential of robot-assisted gait training to achieve clinical benefits for patients with SCI following electrical burns, particularly in improving lower extremity muscle strength, joint range of motion, and gait performance. While the study demonstrated enhancements in lower extremity function and gait parameters, it did not establish the effectiveness or underlying mechanisms of treatment. Specifically, the study lacked an analysis of muscular activity changes and a comparison of lower limb muscle activities or gait analysis during walking with and without robot-assisted gait training in this patient population [34].

In this case, the CVC system of the HAL was utilized. The CVC system detects bioelectric signals on the skin’s surface, regulating the actuators to assist the wearer’s voluntary movements. Integrated into the HAL’s motion support technologies, the control methods enable an interactive biofeedback system, establishing a seamless connection between the brain/nervous system and the device [11]. Locomotor training with HAL is anticipated to enhance operational capacity by reinforcing desirable behavioral patterns in the HAL motion system. Hence, HAL training decreases antagonist muscle activation, resulting in increased agonist muscle activation by coordinating or decreasing high levels of muscle co-contractions and balancing the activities of lower limb muscles, especially during ankle joint movements. Once HAL training reduced this spasticity, the patient may have found it more difficult to walk effectively. Nevertheless, 6 months after the first section, the patient’s gait had improved, with the reduction in spasticity somewhat maintained over time. During the 2nd section, HAL training progressed smoothly, indicating that the patient had adapted to the device, and no decrease in walking speed was observed. Therefore, for patients in similar conditions, a single 10-session HAL training course may be insufficient. Additional sessions and courses may be necessary to fully realize the benefits of HAL training. Moreover, it is important to continue monitoring and studying such cases to better understand how HAL training can be tailored to individual patient needs and how neuroplasticity plays a role in recovery and rehabilitation.

Findings from the gait analysis of this study indicate an increase in walking distance during HAL gait training, particularly in the first section. A deviation from the gait pattern while using double crutches and the HAL gait training was observed, becoming particularly noticeable during the post-10-session evaluation in the first section (Table 1). The increased stride length was notable when using the HAL device, while the cadence remained relatively unchanged. This suggests that the improvement in walking distance and speed was primarily due to the increased stride length. Moreover, with HAL, parameters such as stride length improved, while the CV was reduced, suggesting a stable gait pattern. Since this case involves a chronic condition, the high CV observed in the first section indicates that a longer intervention, such as two sessions, may have been necessary for adaptation. Hence, we hypothesized that the initial difficulties encountered during the first training section were likely due to the patient’s unfamiliarity with HAL training, fatigue, and the need for an adaptation period. Given the significant time elapsed since the patient was struck by lightning, there has been a progression of disuse-related conditions and compensatory alterations in movement patterns, impacting gait kinematics and kinetics. Transitioning from wheelchair use to walking with HAL assistance represents a substantial adjustment for the patient, both physically and neurologically. The gradual improvement observed in the second training section suggested that the patient’s nervous system had begun to adapt, enabling more effective coordination with the HAL device—a process indicative of neuroplasticity often seen in rehabilitation, where patients progressively regain function and coordination. Regarding the reduction in co-contraction during the first section, data from the rectus femoris muscle indicated a decrease in co-contraction (suggesting reduced spasticity). However, this was accompanied by a decrease in the 10 m walking test speed, suggesting that the initial reduction in co-contraction due to HAL training did not immediately translate into improved gait. In fact, the patient may have struggled to develop an effective walking pattern, potentially relying on spasticity to facilitate walking. Before the intervention, the patient exhibited difficulty with excessive knee extension and spasticity in the lower limbs, hindering normal lower limb swing (Video S1). In the second HAL training section, the temporal profile of the angular position of the knee and ankle joints demonstrated greater flexion of the knee joint motion and dorsiflexion angles of the ankle joint during the stance and swing phases than those in the first HAL training section, indicating a change in the early knee-unlocking strategy pattern during the stance phase in the second section compared to that in the first section. In the integrated EMG evaluation, gastrocnemius muscle activation slightly decreased during the stance phase of the second HAL training section. Additionally, in the stance phase, the EMG activity of the rectus femoris decreased in the second HAL training section compared to that in the first. In the swing phase, the rectus femoris EMG activity showed decreased activation between the pre- and post-assessment in both sections. These activities may inhibit abnormal antagonistic EMG muscle activation during ankle joint movements [35]. Previous studies have suggested that HAL training might help correct a patient’s abnormal gait pattern and increase muscle synergy through motor learning [23]. This may be because HAL training altered the involuntary and voluntary EMG waves by reducing antagonistic muscle contraction using computer processing [36].

The patient experienced long-term progression of disuse-related conditions following a lightning strike. Given this background, it is reasonable to assume that the patient required time to adapt to HAL training and initially experienced fatigue. Establishing an effective protocol that includes the appropriate frequency, intensity, and duration of HAL training is essential. Additionally, as this is a case study, a more precise evaluation of the effects of HAL training necessitates the selection of a single-case design [37].

This study was limited by the absence of a comparison group. Hence, further studies involving a higher number of patients are necessary to validate our findings. This case does not merely represent a typical disuse syndrome or reduced walking ability due to muscle weakness; rather, it highlights the clinical significance of achieving outcomes related to neurophysiological coordination and motor learning. Given the disease-specific characteristics and progressive worsening of symptoms observed in this case, there is a need to incorporate innovative perspectives into conventional rehabilitation strategies in the future. This case provides valuable insights into two separate HAL interventions. However, evaluations were only conducted up to 3 months post-intervention, and long-term follow-up was not performed. As the symptoms have been progressively worsening over approximately 40 years, it is imperative to conduct long-term tracking of the patient’s walking ability and daily living activities. Considering the potential risk of regression, it is also crucial to investigate the feasibility of a third HAL intervention as part of a comprehensive long-term preventative strategy for maintaining gait capabilities. Moreover, clinical studies should be conducted to demonstrate the effectiveness of HAL training for progressive spastic paralysis.

## 5. Conclusions

To our knowledge, this is the first study that reported the feasibility and safety of HAL training in patients with spinal cord damage resulting from lightning or electrical trauma. Our results suggest that HAL gait training may inhibit abnormal EMG antagonistic muscle activation, resulting in relatively increased lower-limb joint movements and potentially efficient long-term effects.

## Figures and Tables

**Table 1:** Specific metrics evaluated from the 10 m walking test before the commencement of the first hybrid assistive limb (HAL) session (pre-1 session) and the fifth HAL session (pre-5 session), after the conclusion of all HAL sessions (post-10 session), and 3 months after the second HAL training section. Walking speed, step length, and cadence were measured with HAL (HAL) and without HAL (non-HAL) at the pre-1, pre-5, and post-10 sessions.

Measurements		1st HAL Training Section			2nd HAL Training Section			
		Session			Session			Post-3 Month Evaluation
		1	5	10	1	5	10	
Walking speed (m/min)	non-HAL	8.7	5.2	7.8	10.5	10.2	10.6	10.8
	HAL	4.3	5.9	10.7	11.7	15.3	19	-
Cadence (cycles/min)	non-HAL	25.7	20.8	25.4	30.1	27.6	30.7	30.3
	HAL	12.9	16.7	21.4	25.4	30.7	34.9	-
Stride length (m)	non-HAL	0.34	0.25	0.31	0.35	0.37	0.34	0.36
	HAL	0.33	0.35	0.5	0.46	0.5	0.55	-
WISCI II		11		11	11		12	12
ISNCSCI motor score		76		79	73		77	77
FIM motor score		78		81	82		83	83

## Data Availability

The original contributions presented in the study are included in the article. Further inquiries can be directed to the corresponding authors.

## Supplementary Materials

The following supporting information can be downloaded at: https://www.mdpi.com/article/10.3390/jcm14030967/s1, Video S1: HAL gait training at the pre-1 session, pre-5 session and post-10 session in the first and second HAL training sections. Video S2: The patient’s gait before and after the first and second HAL sections.
